# Immunologic responses to the major allergen of Olea Europaea in local and systemic allergic rhinitis subjects

**DOI:** 10.1186/2045-7022-5-S4-P19

**Published:** 2015-06-26

**Authors:** Paloma Campo, Carmen Rondon, Mayte Villalba, Esther Barrionuevo, Luisa Galindo, Juan Carlos Lopez-Rodriguez, Maria Jose Torres, Cristobalina Mayorga, Miguel Blanca

**Affiliations:** 1 Regional Hospital of Malaga, Malaga, Spain; 2 Regional Hospital of Malaga, UGC Allergy, Malaga, Spain; 3 Faculty of Chemistry, Complutense University of Madrid, Department of Biochemistry and Molecular Biology, Madrid, Spain

## Background

Ole e 1 is one of the major allergens from olive tree pollen. Up to date there are no specific studies that evaluate in depth the in vitro responses to this purified allergen. The goal of the study was to thoroughly evaluate the celullar responses to nOle e 1 in allergic rhinitis (AR) and local allergic rhinitis (LAR) patients with sensitization to olive tree pollen (OL) demonstrated by nasal allergen provocation test (NAPT).

## Methods

Twelve subjects with AR (+NAPT with OL, + skin testing and specific IgE (sIgE) to OL), 12 subjects with LAR (+ NAPT with OL, - skin testing and sIgE to OL), and 12 subjects as control group (CG) (- NAPT, - skin testing and sIgE to OL) were selected. Basophil activation tests (BAT) with OL and nOle e 1, along with dendritic cell (DC) maturation/proliferation studies in response to nOle e 1 stimulation, were carried out in all subjects. Local ethical committee approved the study.

## Results

All AR subjects had positive BAT responses to OL and 10/12 to nOle e1 (83%); 8/12 LAR (66.6%) had a positive BAT with OL and 4/12 (33%) to nOle e1, with only one subject of the control group with a positive BAT to both OL and nOle e1 (8%). DC proliferation and maturation were increased in SAR>LAR>CG but with no significant differences (maturation: 66.7%/57%/50%; proliferation: 40%/20%/0%).

## Conclusion

BAT with OL and nOle e1 in LAR group showed sensitivity between 66.6 and 33%, demonstrating specific basophil activation with pollens in patients with LAR. DC proliferation and maturation were demonstrated in SAR and LAR subjects although with no significant differences with CG.

